# Prevalence of *Neoehrlichia mikurensis* in ticks and rodents from North-west Europe

**DOI:** 10.1186/1756-3305-5-74

**Published:** 2012-07-12

**Authors:** Setareh Jahfari, Manoj Fonville, Paul Hengeveld, Chantal Reusken, Ernst-Jan Scholte, Willem Takken, Paul Heyman, Jolyon M Medlock, Dieter Heylen, Jenny Kleve, Hein Sprong

**Affiliations:** 1 Laboratory for Zoonoses and Environmental Microbiology, National Institute for Public Health and Environment (RIVM), Antonie van Leeuwenhoeklaan 9,, P.O. Box 1, Bilthoven, the Netherlands; 2 Laboratory for Infectious Diseases and Screening, National Institute for Public Health and Environment (RIVM), Antonie van Leeuwenhoeklaan 9, P.O. Box 1, Bilthoven, the Netherlands; 3 National Centre for Monitoring of Vectors (CMV), Netherlands Food and Consumer Product Safety Authority (NVWA), , the Netherlands; 4 Laboratory of Entomology, Wageningen University, Wageningen, The Netherlands; 5 Research Laboratory for Vector Borne Diseases, Queen Astrid Military Hospital, Bruynstraat 1, B-1120, Brussels, Belgium; 6 Medical Entomology & Zoonoses Ecology Group, Microbial Risk Assessment, Emergency Response Department Health Protection Agency, Porton Down, Wiltshire, UK; 7 Evolutionary Ecology Group, University of Antwerp, Groenenborgerlaan 171, B-2020, Antwerpen, Belgium; 8 National Hedgehog and Squirrel Asylum, Amsterdamsestraatweg 29E, Naarden, The Netherlands

**Keywords:** Vector-borne disease, Emerging zoonoses, *Candidatus N. mikurensis*, *I. ricinus*, *Anaplasma phagocytophylum*

## Abstract

**Background:**

*Neoehrlichia mikurensis* s an emerging and vector-borne zoonosis: The first human disease cases were reported in 2010. Limited information is available about the prevalence and distribution of *Neoehrlichia mikurensis* in Europe, its natural life cycle and reservoir hosts. An *Ehrlichia*-like *schotti* variant has been described in questing *Ixodes ricinus* ticks, which could be identical to *Neoehrlichia mikurensis.*

**Methods:**

Three genetic markers, 16S rDNA, gltA and GroEL, of *Ehrlichia schotti*-positive tick lysates were amplified, sequenced and compared to sequences from *Neoehrlichia mikurensis*. Based on these DNA sequences, a multiplex real-time PCR was developed to specifically detect *Neoehrlichia mikurensis* in combination with *Anaplasma phagocytophilum* in tick lysates. Various tick species from different life-stages, particularly *Ixodes ricinus* nymphs*,* were collected from the vegetation or wildlife. Tick lysates and DNA derived from organs of wild rodents were tested by PCR-based methods for the presence of *Neoehrlichia mikurensis.* Prevalence of *Neoehrlichia mikurensis* was calculated together with confidence intervals using Fisher's exact test.

**Results:**

The three genetic markers of *Ehrlichia schotti*-positive field isolates were similar or identical to *Neoehrlichia mikurensis*. *Neoehrlichia mikurensis* was found to be ubiquitously spread in the Netherlands and Belgium, but was not detected in the 401 tick samples from the UK. *Neoehrlichia mikurensis* was found in nymphs and adult *Ixodes ricinus* ticks, but neither in their larvae, nor in any other tick species tested. *Neoehrlichia mikurensis* was detected in diverse organs of some rodent species. Engorging ticks from red deer, European mouflon, wild boar and sheep were found positive for *Neoehrlichia mikurensis*.

**Conclusions:**

*Ehrlichia schotti* is similar, if not identical, to *Neoehrlichia mikurensis. Neoehrlichia mikurensis* is present in questing *Ixodes ricinus* ticks throughout the Netherlands and Belgium. We propose that *Ixodes ricinus* can transstadially, but not transovarially, transmit this microorganism, and that different rodent species may act as reservoir hosts. These data further imply that wildlife and humans are frequently exposed to *Neoehrlichia mikurensis-*infected ticks through tick bites. Future studies should aim to investigate to what extent *Neoehrlichia mikurensis* poses a risk to public health.

## Background

The most prevalent tick-borne infection of humans in the Northern hemisphere is Lyme [1] The same tick species transmitting the etiologic agents of Lyme disease also serve as the vector of pathogens causing tick-borne encephalitis and several forms of rickettsioses, anaplasmoses and ehrlichioses [2]. Members of the family *Anaplasmataceae* are obligatory intracellular bacteria that reside within membrane-enclosed vacuoles. Human ehrlichiosis and anaplasmosis are two closely related diseases caused by various members of the genera *Ehrlichia* and *Anaplasma*. A major difference between these two members is their cellular tropism. *Ehrlichia chaffeensis*, the etiologic agent of human monocytotropic ehrlichiosis (HME), is an emerging zoonosis that causes clinical manifestations ranging from a mild febrile illness to a fulminant disease characterized by multi-organ system failure [3]. *Anaplasma phagocytophilum* causes human granulocytotropic anaplasmosis (HGA), previously known as human granulocytotropic ehrlichiosis [3]. Despite the presence of *Anaplasma phagocytophilum* in questing *Ixodes ricinus* ticks in the Netherlands [4], only one human case has been reported [5]. Seropositivity against anaplasmosis was observed in risk groups, such as foresters and suspected Lyme disease patients, but not in control groups [6]. Still, the incidence of these tick-borne diseases and the associated public health risks remain largely unknown.

A novel candidate species in the family of *Anaplasmataceae*, called *Candidatus Neoehrlichia mikurensis (N. mikurensis)*, was first isolated from wild rats and was also found in *I. ovatus* in Japan [7]. *Neoehrlichia mikurensis* can be distinguished from other genera based on sequence analysis of 16S rDNA, citrate synthase (gltA) and heat shock protein GroEL genes [7]. This recently identified bacterium is detected in several tick species and rodents in different parts of the world under different names [7-11]. The *N. mikurensis* found in *I. ricinus* ticks in Italy has been referred to as *Candidatus Ehrlichia walkerii*[9] and the Ehrlichia species isolated from a rat in China was called “Rattus strain” [12]. Furthermore, a *N. mikurensis* has been described in *I. persulcatus* in Russia [13] and *I. ovatus* from China and Japan [12]. In the US, an Ehrlichia-like organism, closely related to *N. mikurensis,* was previously detected in raccoons. This variant is called *Candidatus Neoehrlichia lotoris*[14]. The Asian *N. mikurensis* isolates showed a 99% similarity based on the 16S rDNA to the *Ehrlichia schotti*. *Ehrlichia schotti* was first described in 1999 in *I. ricinus* in the Netherlands by Leo Schouls and was named after his technician [8]. Later this species was reported in *I. ricinus* in Russia [15] and subsequently in Germany and Slovakia [16]. These findings raised the question whether *Ehrlichia schotti* is the same as *N. mikurensis*.

It is unclear whether *N. mikurensis* poses a risk to public health. Until recently, there were no human infections reported. In 2010, the first case of human *N. mikurensis* infection was reported in a patient from Sweden [17]. In the same year, five other human infections were described in Germany, Switzerland and the Czech Republic [18]. More recently, a canine infection was reported in Germany [19]. The symptoms described in all of these cases were generally non-specific and usually seen in any other ordinary inflammation reaction (Table 1). These reported cases of human infections imply that re-evaluation is needed regarding the pathogenesis of this species. All but one case that have been described so far have occurred in patients who were immuno-compromised. The non-specificity of the reported symptoms, poor diagnostic tools and the lack of awareness of public health professionals could explain the absence of (reported) patients.

**Table 1:** **Reported human cases of*N. mikurensis*infection (until October 2011)**

**Location**	**Case**	**Symptoms and clinical signs**	**Ref.**
Germany	Male, 69yr Immunosuppressive therapy	Episodes of fever, nonproductive cough, left thoracic pain, vein thrombosis, hypochromic anemia, reduced numbers of leukocytes, decreased percentage of lymphocytes, increased proportion of monocytes and elevated levels of CRP, microbiological analysis were negative.	[18]
Germany	Male, 57yr Previously healthy	Headaches, fever, intracerebral and subarachnoid hemorrhage, aneurysm, elevated CRP, pulmonary infiltration, microbiological analyses were negative, elevated infection parameters. Patient died from septic multi-organ failure.	[18]
Sweden	Male, 77yr Chronic lymphocytic leukemia	Transitory ischemic attack, hemolytic anemia, fever, erysipelas-like rash, transitory weakness of the left side of face and arm, hemolytic anemia, thrombocytopenia, thrombosis, pulmonary infiltration, increased proportion of monocytes and elevated levels of CRP, blood and other cultures were negative	[17]
Switzerland	Male, 61yr CABG surgery	Malaise, fever, moderate dyspnea, elevated leukocytes/neutrophils, elevated CRP, microbiological analysis were negative	[20]
Czech Republic	Female 55 yr Mantle Cell Lymphoma	Spiking fever, myalgias, arthralgias, erthema nodosum, elevated CRP, blood-, urine culture and pharyngeal swabs were negative. Antinuclear-, antinucleolar antigens and rheumatoid factor screens were negative.	[21]
Czech Republic	Male, 58yr Liver transplantation and splenectomy	Spiking fever, extreme fatigue, joint pain, skin erythema, painful and stiffened subcutaneous veins, mild leukocytosis and elevated CPR, blood and urine cultures and pharyngeal swab were negative.	[21]

In this study we aim to investigate (i) whether *Ehrlichia schotti* is similar to the described *N. mikurensis* family, (ii) the distribution and prevalence of *N. mikurensis* in the Netherlands, Belgium and the UK, (iii) possible transmission routes of *N. mikurensis* in non-experimental settings and (vi) its putative mammalian hosts.

## Methods

### Collection, identification and DNA extraction of ticks

Questing *I. ricinus* from all stages and *Dermacentor reticulatus* adults were collected in 2009 and 2010 by flagging the vegetation at geographically different locations in the Netherlands and Belgium. Ticks collected in the UK and Vrouwenpolder (NL) have been described before [22]. For global geographic location, see Additional file 1: Figure S1. Questing *I. arboricola* were collected from bird nests in two different areas in Belgium. *Ixodes hexagonus* feeding on hedgehogs were collected in a hedgehog-shelter in 2010. *Ixodes ricinus* feeding on red deer (*Cervus elaphus*), European mouflon *(Ovis orientalis musimon*), wild boar (*Sus scrofa*) and sheep (*Ovis aries*) were collected. All the collected ticks were immersed in 70% alcohol and stored at −20°C until the DNA extraction. Based on morphological criteria, tick species and stages were identified to species level, with stage and sex recorded [23]. In doubtful cases, sequencing of tick mitochondrial 16S rDNA confirmed the tick-species [24]. DNA from questing ticks was extracted by alkaline lysis [4]. DNA from engorged ticks was extracted using the Qiagen DNeasy Blood & Tissue Kit according to the manufacturer’s manual (Qiagen, 2006, Hilden; Germany) following the manufacturer’s protocol for the purification of total DNA from ticks.

### Preparation of DNA lysates from wild rodents

Longworth traps (Bolton Inc., UK), baited with hay, apple, carrot, oatmeal and mealworm were used to capture different species of rodents and insectivores at 7 different locations in the Netherlands between 2007 and 2010. Animals were anaesthetized with isoflurane and euthanized by cardiac puncture. Serum was collected and stored at – 20°C. Spleen, liver, kidney, brain and other organs were collected and frozen at −80°C. DNA was extracted using the Qiagen DNeasy Blood & Tissue Kit according to the manufacturer’s manual (Qiagen, 2006, Hilden; Germany). All animals were handled in compliance with Dutch laws on animal handling and welfare (RIVM/DEC permits).

### Polymerase chain reactions

Polymerase chain reaction (PCR) amplifications were performed in a *P*x2 Thermal Cycler (Thermo Electron Corporation, Waltham, Massachusetts, USA). The presence of *Ehrlichia schotti* in questing *I. ricinus* was studied by Reverse Line Blotting as described [25]. Fragments of the 16S rDNA, citrate synthase gene gltA, and the chaperonin GroEL of ehrlicial species were amplified from tick lysates and rodent tissue samples using novel primers and primers that were previously described (Table 2). Amplification of gltA and GroEL were both done in 50 μl reaction volumes containing 5 μl template DNA. GltA DNA was amplified using a final concentration of 800 nM of each primer, NMik fo-gltA and NMik re-gltA with the following PCR program, 15 min at 95°C, 40 cycles each consisting of 30 sec at 94°C, 25 sec at 53°C, and 10 min at 72°C. GroEL DNA was amplified using, 500 nM of each primer NMik fo-groEL and NMik re-groEL. The PCR program used is as followed: 15 min at 95°C, 40 cycles each consisting of 30 sec at 94°C, 75 sec at 49°C, and 10 min at 72°C. The nested reaction was carried out at the same temperature as the first reactions; only 25 cycles were carried out with 1 μL of the first amplification product. The HotStarTaq Polymerase Kit (Qiagen) was used for all PCR experiments. PCR products were detected by electrophoresis in a 1.5% agarose gel stained with SYBR gold (invitrogen).

**Table 2:** **Primers used for amplification and sequencing of gltA and GroEL genes of*N. mikurensis*, and the amplification of the Msp2 gene of*A. phagocytophilum***

**Gene**	**Name**	**Type**	**Sequence**	**Reference**
gltA	NMik fo-gltA	Primer (forward)	5’-aagtgcatgctttgctacatt-‘3	This study
gltA	NMik re-gltA	Primer (reverse)	5’-tcatgatctgcatgtaaaataaat-‘3	This study
GroEL	NMikGroEL-F2a	Primer (forward)	5’-ccttgaaaatatagcaagatcaggtag-‘3	This study
GroEL	NMikGroEL-R2b	Primer (reverse)	5’-ccaccacgtaacttatttagtactaaag -‘3	This study
GroEL	NMikGroEL-P2a	Probe (RED)	5’-RED-cctctactaattattgctgaagatgtagaaggtgaagc-BHQ2-‘3	This study
GroEL	NMik fo-groEL	Primer (forward)	5’-gaagyatagtytagtatttttgtc-‘3	[18]
GroEL	NMik re-groEL	Primer (reverse)	5’-ttaacttctacttcacttgaacc-‘3	[18]
GroEL	NMik seq1groEL	Primer (reverse)	5’-acatcacgcttcatagaaag-‘3	[18]
GroEL	NMik seq2groEL	Primer (forward)	5’-aaaggaattagtattagaatcttt-‘3	[18]
GroEL	NMik seq3groEL	Primer (forward)	5’-aatatagcaagatcaggtagac-‘3	[18]
GroEL	NMik seq4groEL	Primer (reverse)	5’-cttccattttaactgctaattc-‘3	[18]
Msp2	ApMSP2F	Primer (forward)	5’-atggaaggtagtgttggttatggtatt-‘3	[26]
Msp2	ApMSP2R	Primer (reverse)	5’-ttggtcttgaagcgctcgta-‘3	[26]
Msp2	ApMSP2P	Probe (FAM)	5’-FAM-tggtgccagggttgagcttgagattg-BHQ1-‘3	[26]

Primers were either identical to or slightly modified from the primers described in the reference papers.

### Multiplex real-time PCR

Oligonucleotide primer and probe sequences were designed to be specific for the *N. mikurensis* GroEL gene using Visual OMP DNA (Software, Inc., Ann Arbor, USA). Primer sequences for the *N. mikurensis* GroEL gene were NMikGroEL-F2a and NMikGroEL-R2b and generated a 99-bp fragment which was detected with the NMikGroEL-P2a TaqMan probe (Table 2). Sequences were evaluated on the basis of the following criteria: predicted cross-reactivity with closely related organisms, internal primer binding properties for hairpin and primer-dimer potential, length of the desired amplicon, G-C content, and melting temperatures (*T*_*m*_s) of probes and primers. The specificity of the *N. mikurensis* GroEL primers for *N. mikurensis* in the multiplex real-time PCR assay was tested with DNA extracted from the following microorganisms: *Rickettsia rickettsiiAnaplasma phagocytophilumR. helveticaBartonella henselaeEhrlichia canisB. afzeliiB. garinii, B. sensu stricto, Babesia microti, Candidatus Midichloria mitochondrii* and tick lysates containing *Wolbachia species*[22,25] None were amplified. Random samples of tick lysates which were *N. mikurensis*-positive in the Q-PCR were routinely confirmed by conventional PCR using NMik fo-gltA and NMik re-gltA primers, followed by DNA sequencing.

### Optimized conditions for multiplex PCR

PCR was performed in a multiplex format with a reaction volume of 20 μl, using the iQ Multiplex Powermix (Bio-Rad Laboratories, Hercules, USA), in the LightCycler 480 Real-Time PCR System (F. Hoffmann-La Roche, Basel, Switzerland). Final PCR reaction concentrations were 1x iQ Powermix, primers ApMSP2F and ApMSP2R at 250 nM each, probe ApMSP2P-FAM at 125 nM, primers NMikGroEL-F2a and NMikGroEL-R2b at 250 nM each, probe NMikGroEL-P2a-RED at 250 nM, and 3 μl of template DNA. Cycling conditions were: 95°C for 5 min, followed by 60 cycles of a 5 sec denaturation at 95°C followed by a 35 sec annealing-extension step at 60°C. Ticks lysates were considered positive if the Ct-value of a proper sigmoid curve was maximally three cycles more than the highest dilution of the positive control sample.

For each PCR and real-time multiplex PCR, positive, negative controls and blank samples were included. A 10^-3^ to 10^-5^ dilution of a mixture of sequencing-confirmed *N. mikurensis*-positive tick lysates were used as positive controls. In order to minimize contamination, the reagent setup, the extraction and sample addition, and the real-time PCR as well as sample analysis were performed in three separate rooms, of which the first two rooms were kept at positive pressure and had airlocks.

### DNA sequencing and genetic analysis

PCR amplicons were sequenced using the described primers (Table 2) and the BigDye Terminator Cycle sequencing Ready Reaction kit (Perkin Elmer, Applied Biosystems). All sequences were confirmed by sequencing both strands. Sequences were compared with sequences in Genbank using BLAST after subtraction of the primer sequences (http://www.ncbi.nlm.nih.gov/genbank/). The collected sequences were assembled, edited, and analysed with BioNumerics version 6.5 (Applied Maths NV, Sint-Martens-Latem, Belgium). Resulting sequences were aligned with those from related organisms in Genbank. Phylogenetic analyses of the sequences and related organisms were conducted using the BioNumerics program using the neighbour-joining algorithm with Kimura's two-parameter model. Bootstrap proportions were calculated by the analysis of 1000 replicates for neighbour-joining trees. DNA sequences are available upon request.

## Results

### Comparison of Ehrlichia schotti with N. mikurensis

**Figure 1:**
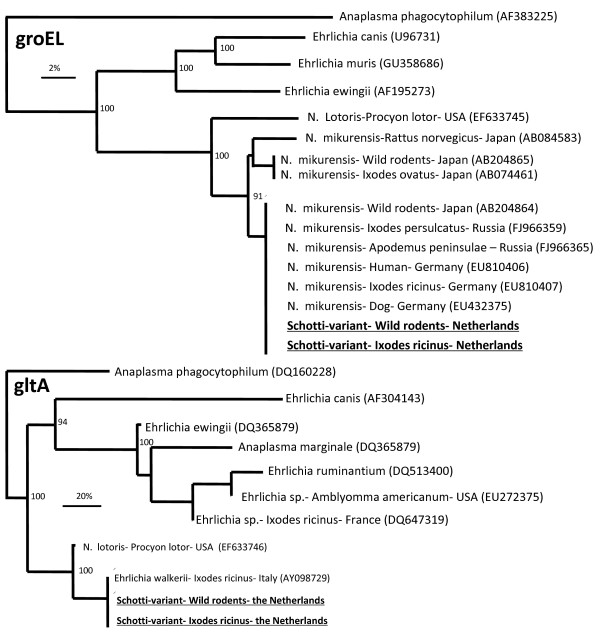
**Phylogenetic tree of the GroEL (top) and gltA (bottom) of different Anaplasma and Ehrlichia species and their relation with*N. mikurensis*and related species.***Ehrlichia schotti/N. mikurensis* sequences from *I. ricinus* (n = 26) and rodents (n = 11), which were generated in this study are depicted in bold. Other GroEL and gltA sequences were taken from Genbank. Their accession numbers are shown between brackets. The evolutionary distance values were determined by the method of Kimura, and the tree was constructed according to the neighbour-joining method. Bootstrap values higher than 90%, are indicated at the nodes.

Twenty-three tick lysates that were previously tested positive for the presence of *Ehrlichia schotti* by PCR and Reverse Line Blotting [5,9,27] were amplified by PCR on the three loci 16S rDNA, gltA and GroEL using primers specific for *N. mikurensis* (Table 2)*.* Amplicons of all three partial genes were obtained in 21 cases. None of these three loci were successfully amplified in 15 *Ehrlichia schotti*-negative ticks. The PCR products of all three loci were sequenced and compared with each other and with *N. mikurensis* sequences available in Genbank. All the *Ehrlichia schotti* sequences were identical to each other on the three loci, 16S rDNA, gltA as well as the GroEL. The 1740 base pairs of the 16S rDNA sequences from the *Ehrlichia schotti* were 99.6% to 100% similar to the *N. mikurensis* sequences and the *Candidatus Ehrlichia walkerii* sequence in Genbank (Table 3). The 233 base-pair fragment of the gltA sequences from the *Ehrlichia schotti* were identical to the *Candidatus Ehrlichia walkerii* gltA sequence (Table 3). The 1238 base-pairs of the GroEL isolates amplified from the tick lysates showed a 94.3% and 95.5%, 98.7% and 100% (AB084583 and AB074461, EF633745 and FJ966365) match with the *N. mikurensis* GroEL sequences in Genbank, respectively. Phylogenetic analyses of the gltA and GroEL sequences showed that the *Ehrlichia schotti* clustered with *N. mikurensis* isolates, but not with *A. phagocytophilum* or any of the Ehrlichia species present in Genbank (Figure 1).

**Table 3:** **Members of the*N. mikurensis*group are distinguished from other genera based on sequence analysis of 16S rDNA, citrate synthase (gltA) and heat shock protein GroEL genes**

**Country (Ref.)**	**Species**	**Named**	**Gene**	**AccessionN**	**Similarity**
Netherlands [8]	*I. ricinus*	*Ehrlichia-like ‘schotti variant’*	16S	AF104680	100%
Russia [15]	*I. ricinus* *I. persulcatus*	*Ehrlichia-like ‘schotti variant’*	16S	AF104680	100%
Germany [16]	*I. ricinus*	*Ehrlichia-like ‘schotti variant’*	16S GroEL	AF104680 EU810407	100% 100%
Italy [9]	*I. ricinus*	*C. Ehrlichia walkerii*	16S GltA	AY098730 AY098729	100% 100%
Italy [28]	*I. ricinus*	*C. Ehrlichia walkerii*	16S GltA	AY098730 AY098729	100% 100%
China [12]	*Rattus norvegicus*	*Ehrlichia-like ‘Rattus variant’*	16S	AY135531	98.9%
Japan [7]	*Rattus norvegicus* *I. ovatus*	*C. N. mikurensis* (TK4456 and IS58)	16S GroEL	AB084582 AB074460 AB084583 AB074461	99.1% 99.4% 94.3% 95.5%
USA [14]	*Procyon lotor*	*Ehrlichia-like organism*	16S	AY781777	99.8%
Japan [29]	*A. argenteus* *A. speciosus and Eothenomys.smithii*	*C. N. mikurensis* (FIN686 and Nagano21)	16S	AB196304 AB196305	99.5% 99.6%
Russia [13]	*I. persulcatus*	*Ehrlichia-like ‘schotti variant’*	16S	AF104680	100%
Italy [11]	*C. glareolus*	*C. N. mikurensis*	16S	AB213021	99.6%
Russia [30]	*M. rossiaemeridionalis* *I. persulcatus*	*C. N. mikurensis*	16S	EF445398	100%
USA [31]	*Procyon lotor*	*C. N. lotoris* (RAC413)	16S GroEL GltA	EF633744, EF633745 EF633746	97.8% 98.7% 79.5%
Slovakia [32]	*I. ricinus*	*C. N. mikurensis*	16S	AB196305	99.7%
Russia [30]	*I. persulcatus* *A. peninsulae*	*C. N. mikurensis*	16S GroEL	FJ966364 FJ966363 FJ966366 FJ966365	99.6% 100% 98.7% 98.7%
Germany [18]	Human	*C. N. mikurensis*	16S, GroEL	EU810404 EU810406	99.9% 100%
Switzerland [20]	Human	*C. N. mikurensis*	16S GroEL	GQ501089 HM045824	100% 98.9%
Germany [19]	Dog	*C. N. mikurensis*	GroEL	EU432375	100%

This strain has been reported in different parts of the world under diverse nominations. The similarity of these isolates with *N. mikurensis* isolates present in Dutch ticks isolates were calculated.

### Prevalence and distribution of N. mikurensis

In order to estimate the prevalence and distribution of *N. mikurensis* in North-West Europe, questing *I. ricinus* nymphs (~88%) and adults (~12%) were tested using a Q-PCR for the simultaneous detection of *N. mikurensis* and *A. phagocytophilum.* In all 12 study-areas in the Netherlands, *N. mikurensis* was detected with a prevalence varying from 1% to 16% (Table 4). Ticks from one study area (Duin en Kruidberg) were tested in two consecutive years. In 2009, 16% of the questing nymphs and adults *I. ricinus* were infected with *N. mikurensis.* The prevalence of *N. mikurensis* in questing ticks decreased to 8% in 2010. *Neoehrlichia mikurensis*-positive *I. ricinus* ticks were found in two out of three regions in Belgium. A fraction of the ticks from the *N. mikurensis*-negative area (Brussels) were positive for *A. phagocytophilum,* which was comparable to other regions (data not shown), indicating that the processing and testing of ticks from this area was not affecting the outcome of the results. The results for the *A. phagocytophilum* will be published elsewhere. To determine whether *N. mikurensis* is present in the UK, 338 *I. ricinus* and 63 *D. reticularis* ticks from a previous study were tested [27]. These ticks were collected at 7 dispersed study areas in the UK and were partially caught by blanket dragging and removed from wildlife, pets and humans. *Anaplasma phagocytophilum,* but not *N. mikurensis*, was detected in these tick lysates.

**Table 4:** **The prevalence and distribution of*N. mikurensis*in questing*I. ricinus*in the Netherlands and Belgium**

**Location**	**Tested (n)**	**Positive (n)**	**Prevalence (%)**
Boswachterij Hardenberg	90	7	8% (3-15%)
Dintelse Gorzen	122	9	7% (3-14%)
Drents-Friese Wold	29	1	3% (0-18%)
Duin en Kruidberg (2009)	320	52	16% (12-21%)
Duin en Kruidberg (2010)	137	11	8% (4-14%)
Hoog Soeren	217	3	1% (0-4%)
Kop van Schouwen	238	23	10% (6-14%)
Denekamp	104	4	4% (1-10%)
Pyramide van Austerlitz	270	32	12% (8-16%)
Rijk van Nijmegen	53	1	2% (0-10%)
Ulvenhoutse bos	8	1	13% (0-53%)
Vijlenerbos	328	10	3% (2-5%)
Vrouwenpolder	86	6	7% (3-15%)
Brussel-area, (Sonian forest), (Belgium)	153	0	0% (<2%)
Vlaanderen-area (Belgium)	114	3	3% (1-8%)
Wallonië-area (Belgium)	106	3	3% (1-8%)
***Total of all ticks***	***2375***	***166***	***7% (6-8%)***
***Average of all areas***	***15***	***14***	***6%***

Confidence intervals (95%), which were calculated using Fisher's exact test, are between brackets. The average of all areas was calculated by average of all prevalence’s excluding Duin en Kruidberg 2009.

### Role of ticks in the transmission of N. mikurensis

Transovarial (vertical) transmission has been implicated for Rickettsia [33] and Anaplasma [34], but not for Ehrlichia species [35]. Whether *N. mikurensis* is transmitted transovarially in *I. ricinus* has not been investigated so far. The prevalence of *N. mikurensis* was determined in 55 pools of 5 questing *I. ricinus* larvae from Vrouwenpolder, where nymphal and adult ticks were found to be positive for *N. mikurensis* (Table 4). None of the 55 pools were *N. mikurensis-*positive (Table 5)*.* Some of the pools were positive for *A. phagocytophilum*, approving the used methodology. The prevalence of *N. mikurensis* in questing *I. ricinus* nymphs was ~7%, whereas the prevalence in adult ticks was ~ 11% (Table 5). No significant differences were observed in the prevalence between questing male and female *I. ricinus* ticks. To investigate the role of other tick species in the transmission of *N. mikurensis: Dermacentor reticulatusI. hexagonus* and *I. arboricola* were analysed for the presence of *N. mikurensis* (in the multiplex real-time PCR). None were found positive (Table 6). Again, some were found positive for the *A. phagocytophilum* msp2 gene (data not shown), indicating that there is no significant inhibition within these samples.

**Table 5:** **Prevalence of*N. mikurensis*in questing*I. ricinus*, divided by lifecycle stage**

**Stage**	**Tested (n)**	**Positive (n)**	**Prevalence (%)**
Larvae	55*	0	0% (<1%)
Nymph	2003	137	7% (6-8%)
Female	92	10	11% (5-20%)
Male	173	19	11% (7-17%)

*Pools of 5 larvae. 95% Confidence intervals were calculated using Fisher's exact test and are between brackets.

**Table 6:** ***D. reticularis, I. hexagonus*and*I. arboricola*tested in the multiplex real-time PCR for the*N. mikurensis.***

**Tick species**	**Tested (n)**	**Positive (n)**	**Prevalence (%)**
*I. arboricola*	79	0	0% (<5%)
*I. hexagonus*	169	0	0% (<2%)
*Dermacentor reticulatus*	177	0	0% (<2%)

Confidence intervals (95%), which were calculated using Fisher's exact test.

### Potential reservoir hosts of N. mikurensis

To investigate the possible mammalian hosts for *N. mikurensis*, 79 spleen samples of different wild small mammals were tested by (nested)-PCR for the presence of gltA and GroEL (Table 7). PCR-positive samples were sequenced to confirm the presence of *N. mikurensis.* Both the GroEL and gltA sequences isolated from spleen were identical to the *N. mikurensis* sequences found in the questing ticks in the Netherlands (Figure 1). Spleen samples from *Apodemus sylvaticus*, *Microtus arvalis* and *Myodes glareolus* were *N. mikurensis*-positive. After the spleen was found positive, other organs (kidney, liver and brain) were also tested for *N. mikurensis*. All the tested organs were positive.

**Table 7:** **Spleens of wild rodent and insectivore species were tested by PCR and sequencing using*N. mikurensis*specific primers**

**Rodent species**	**Tested (n)**	**Positive (n)**
*Apodemus flavicollis*	2	0
*Apodemus sylvaticus*	23	5
*Crocidura russula*	5	0
*Microtus arvalis*	8	2
*Myodes glareolus*	35	4
*Sorex araneus*	6	0
***Total***	***79***	***11***

Whether other mammals in the Netherlands are reservoir hosts is difficult to address, due to the protective status of these animals. An animal can be considered a potential reservoir host when the prevalence of *N. mikurensis* in ticks feeding on this animal is significantly higher than the prevalence in questing ticks. This is for example the case for *Anaplasma phagocytophilum*[36-40]. *I. ricinus* feeding on red deer (*Cervus elaphus*), European mouflon (*Ovis orientalis musimon*), wild boar (*Sus scrofa*) and sheep (*Ovis aries*) were tested by multiplex real-time PCR. The prevalence of *N. mikurensis* in feeding ticks was comparable to the prevalence in questing ticks (Table 8).

**Table 8:** ***I. ricinus*adults feeding on animals living in nature reserve areas in the Netherlands were tested by multiplex real-time PCR for the presence of*N. mikurensis***

**Ticks from**	**Ticks** **tested (n)**	**Ticks** **Positive (n)**	**Prevalence in ticks (%)**	**Animals** **tested (n)**	**Animals with** **positive ticks (n)**
*Cervus elaphus*	409	26	6% (4-9%)	17	10
*Sus scrofa*	48	4	8% (2-20%)	8	2
*Ovis aries*	264	33	13% (9-17%)	24	13
*Ovis orientalis musimon*	233	10	4% (2%-8%)	18	4

## Discussion

Recently, six human and one canine case of *N. mikurensis* infection were reported in different locations in Europe. These reports advocate a re-assessment of the occurrence of this microorganism in questing ticks. Schouls and colleagues described an Ehrlichia-like organism (*Ehrlichia schotti)* in Dutch ticks [8]. In our study, the three genetic markers 16S rDNA, gltA and GroEL of *Ehrlichia schotti*-positive field isolates turned out to be similar and identical to DNA sequences available from *N. mikurensis*. Thus, *Ehrlichia schotti* and *N. mikurensis* are most likely one and the same species. Previous findings on *E. schotti* can be interpreted as findings on *N. mikurensis*. Thus, *N. mikurensis* has already been present in the Netherlands in 1999 [25,41]. Furthermore, 11% of 289 engorged *I. ricinus* removed from humans were *N. mikurensis-*positive, indicating that the Dutch population is being exposed to ticks infected with *N. mikurensis*[42]. Remarkably, human and animal cases of *N. mikurensis* infection in the Netherlands have not yet been described.

The development of a Q-PCR specific for *N. mikurensis* allowed us to test significant numbers of ticks without having to perform the labour-intensive Reverse-Line blotting. These analyses showed that the *N. mikurensis* is present in vegetation ticks throughout the Netherlands and Belgium. No *N. mikurensis*-positive ticks were found in one location in Belgium. One possible explanation is that this location in the Brussels-area is exceptional due to its reduced fauna and flora caused by human interference. This forest in the Brussels-area is also highly fragmented because of a railroad and several major motorways that run through the forest. Several parts of it can be ecologically considered ‘islands’, which could -through isolation of mammal and tick populations- explain the absence of the pathogen in this forest. More ticks of this unique area need to be tested in order to address this hypothesis. *Neoehrlichia mikurensis* was also not detected in ticks from the UK. This could indicate that these species have not (yet) been established on this island.

The overall prevalence of *N. mikurensis* in questing nymphs and adults is approximately 7%. From the public health point of view, it indicates that a significant proportion of people contracting a tick bite are exposed to *N. mikurensis.* Transmission of the *N. mikurensis* in ticks appears to occur horizontally rather than vertically. None of the tested larvae were found positive, even though the prevalence of nymphs is approximately 7% and 11% for adults. Other tick species, with more restricted host preference than *I. ricinus*, were also tested for the presence of *N. mikurensis. Dermacentor reticulatus, I. hexagonus* and questing *I. arboricola* were found negative. The data indicate that these tick species probably play insignificant roles in the transmission of *N. mikurensis.* In contrast, *I. ricinus* can be considered as its main vector in the Netherlands and Belgium.

A potential group of reservoir hosts for *N. mikurensis* are wild rodents. Indeed, spleen samples and other organs (kidney, liver and brain) of some rodent species turned out to be *N. mikurensis*-positive, which indicates a systemic infection of these rodents with *N. mikurensis.* The *N. mikurensis* isolates from ticks and wild rodents (Table 7) were genetically identical, indicating that rodents are potential reservoir hosts [43]. However, the reservoir potential of rodents can only be by xenodiagnosis or experimental infection. The prevalence of *N. mikurensis* in *I. ricinus* ticks feeding on red deer, European mouflon, wild boar and sheep were comparable to the prevalence in questing ticks. From these prevalence data, it was not possible to infer the role of these animals in the transmission of *N. mikurensis.* However, it is clear that these animals are being exposed to the *N. mikurensis* through tick bites. Further experiments are necessary to determine whether there are other mammalian reservoirs than wild rodents.

## Conclusions

Although human infection of the *N. mikurensis* has not been reported in the Netherlands, it is unclear to what extent *N. mikurensis* poses risks to public health. The symptoms described in all of the *N. mikurensis* infection cases were generally non-specific and usually seen in any other ordinary inflammatory reaction. What’s more, most of the Ehrlichia infections are known to be either asymptomatic or mild, self-limiting diseases [3]. In other words, infection can occur without causing disease. So far, diagnosis has relied only on PCR amplification of the *N. mikurensis*. The lack of serological tests makes diagnosis particularly difficult. Against these backdrops, the actual incidence of human infection with Ehrlichia is likely to be much higher than currently reported in Europe. Thorough surveillance and improvement of diagnostic tools will probably increase the number of identified human cases, and consequently provide more insight in the public health relevance of *N. mikurensis.*

## **Competing interests**

The authors declare that they have no competing interests.

## **Authors’ contributions**

CR, EJS, WT, PH, DH, JK and JM organized and participated in the fieldwork for the collection and determination of wildlife samples and ectoparasites. SJ and MF developed the laboratory tests for the simultaneous detection of *N. mikurensis* and *A. phagocytophilum*. SJ, MF and MF carried out the laboratory preparation and testing of all the samples. SJ performed the sequencing and sequence analyses. SJ and HS drafted the manuscript and wrote the final version. HS organized and planned the study. All authors read and approved the final manuscript.

## Supplementary Material

Additional file 1: Figure S1 Geographical distribution of locations (rounds) or global areas (stars) of questing *I. ricinus* tested positive (red) or negative (green) for *N. mikurensis* in The Netherlands and Belgium*.* Exact coordinates of geographical locations are available upon request. Click here for file
