# Supplementary material for: Prevalence of Neoehrlichia mikurensis in ticks and rodents from North-west Europe
Source: Parasit Vectors. 2012 Jul 12;5:74. doi: 10.1186/1756-3305-5-74 (PMC3395572; doi:10.1186/1756-3305-5-74)

Supplementary Figure 1: Geographical distribution of locations (rounds) or global areas (stars) of questing *I. ricinus* tested positive (red) or negative (green) for *N. mikurensis* in The Netherlands and Belgium*.* Exact coordinates of geographical locations are available upon request.


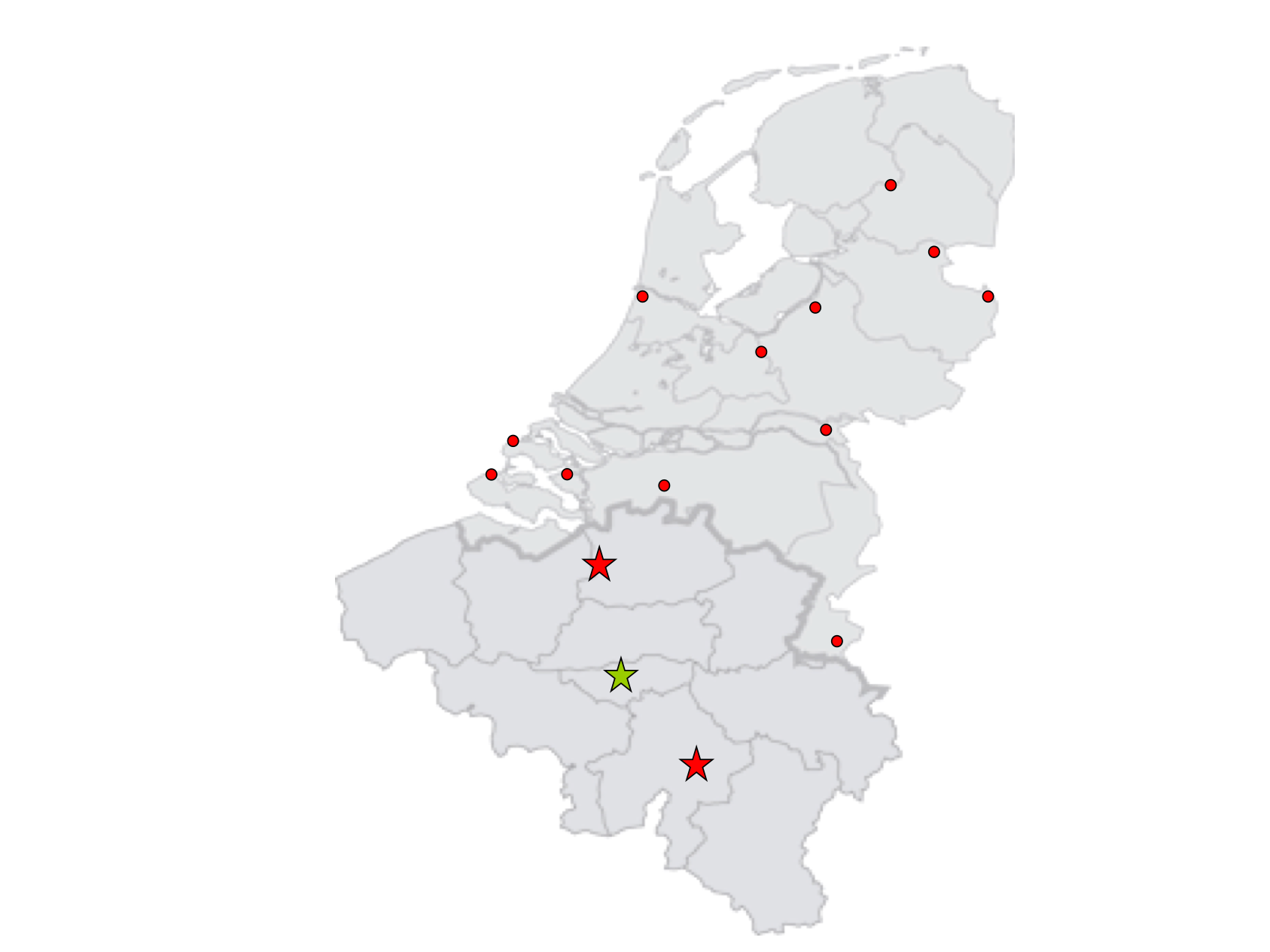

Supplement: Additional file 1: Figure S1 — Geographical distribution of locations (rounds) or global areas (stars) of questing I. ricinus tested positive (red) or negative (green) for N. mikurensis in The Netherlands and Belgium. Exact coordinates of geographical locations are available upon request. [file 1756-3305-5-74-S1.docx]
